# Implementation of 3D printing technology for complex spine revision cases that require multilevel anterior spinal support: Over 5-year experience in six cases and costs assessment

**DOI:** 10.1016/j.bas.2026.105929

**Published:** 2026-01-06

**Authors:** J. Magré, M.S. Ramselaar, K. Willemsen, H. Weinans, T.P.C. Schlösser, M.C. Kruyt

**Affiliations:** a 3D Lab, Division of Surgical Specialties, University Medical Center Utrecht, 3584 CX, Utrecht, the Netherlands; b Department Orthopedics, University Medical Center Utrecht, 3584 CX, Utrecht, the Netherlands; c Department of Biomechanical Engineering, Delft University of Technology, 2628 CD, Delft, the Netherlands; d Department of Biomechanical Engineering, Twente University, 7522 NB, Enschede, the Netherlands

**Keywords:** Spine, Custom-made, Prosthesis, 3D printing, Allograft, Anterior fusion

## Abstract

**Introduction:**

The anterior column of the spine is crucial for stability. In a dystrophic spine, the loss of multisegmental anterior spinal support can have devastating consequences. Since posterior instrumentation alone cannot take over the weight bearing capacity of the anterior column, structural anterior support must be created. Long bone struts are at risk for failure of engraftment and pseudoarthrosis. Patient-specific anterior support using 3D printing technology may be a better solution in these patients.

**Research question:**

Are patient-specific approaches using 3D printing technology a viable treatment option for multilevel anterior spinal support?

**Material and methods:**

Five patients received a custom-made anterior paravertebral titanium spinal strut prosthesis; one patient received a 3D shaped structural allograft. Cost assessment was made based on hours spent and production costs. Clinical outcomes were extracted from the medical records.

**Results:**

All six implantations went uneventful with adequate fit of the prostheses and allograft. The mean surgery time was 219 min, and mean blood loss was 850 ml. No implant subsidence or loosening occurred during follow-up (0.5–8 years). Complications observed were partial bronchial compression in one patient and a postoperative infection in another. The first cases were most costly due to the hours spent on design and regulatory compliance. These costs declined for subsequent cases.

**Discussion and conclusion:**

Custom-made prostheses appear to be a viable treatment option for multi-level anterior spinal support. No implant failure was observed up to 8 years postoperative. Close collaboration between an in-house 3D lab and the surgical team was essential for implementing custom-made prosthesis in clinical care.

## Highlights

• 3D printed spinal prostheses restore anterior support in dystrophic spines.
• 3D printing enables tailored anterior spinal reconstruction.
• Close collaboration between surgeons and the 3D Lab was instrumental for success.

## 1 Introduction

The anterior column of the spine, composed of vertebrae and intervertebral discs, plays a critical role in weight bearing and ambulation (Gracovetsky, 1986). Diseases that can cause loss of anterior support over multiple levels include lytic tumors, spondylitis, vanishing bone disease and spinal dystrophy by neurofibromatosis (NF1) (Ciftdemir et al., 2016; Nikolaou et al., 2014; Tsirikos et al., 2005; Durrani et al., 2000). Loss of anterior support can lead to progressive deformity and mechanical instability. Eventually leading to a collapse of the spine with devastating consequences.

In general, posterior instrumentation alone cannot take over the lack of anterior support and will eventually fail due to overloading or fatigue. Traditionally, restoration of the anterior column is performed with (vascularized) fibular or rib autograft (Saraph et al., 2005). Although these grafts offer advantages in terms of availability and relative ease of harvest, they also present drawbacks, such as long operative times, donor site morbidity, risk for early dislocation and failure of graft incorporation with non-union rates ranging from 0 % to 54 % (Ikard, 2006; Riasa and Kawilarang, 2023). Furthermore, in cases of dystrophy and/or dural ectasias, the affected vertebrae cannot be used as a foundation for engraftment and should be bypassed.

To overcome these challenges, custom-made prostheses that are specifically designed to create immediate stability and bypass the affected area, could offer a relatively simple and effective solution in restoring anterior support. Compared to autograft struts there is no donor site morbidity, no vascular anastomosis is needed, there is no engraftment issue and there are no limitations in graft length. However, this approach has been rarely reported to date, and long-term effects are lacking.

Over the past five years, we developed an in-house pathway for custom-made 3D-printed titanium anterior spinal strut prostheses for exceptional complex cases with multilevel lack of anterior support. In accordance with EU (Medical Device Regulations, MDR) regulations, the implants are co-designed by an engineer and a surgeon in our in-house 3D Lab, with outsourced final production of the implant. This in-house development pathway is necessary because commercial providers consider these cases to pose excessive legal liability. We designed a prosthesis that can be inserted through standard approaches, allows for incorporation of bone into the porous ends of the implant, can be fixated to the spine with preplanned strategic screws, and has a massive titanium stem to minimize the risk of fatigue failure in time (Willemsen et al., 2019). The first application of this prosthesis was reported 6 years ago (Willemsen et al., 2019). Since then, we further improved the technique and established a regulatory pathway for in-house development of medical devices. A total of five patients have been treated according to this principle, and a 6th pediatric patient with severe early onset scoliosis was treated with a 3D shaped allograft. The intended purpose of these prostheses was to provide anterior support, without aiming to correct the spinal deformity.

The current manuscript evaluates our clinical and surgical experience, along with over five-year follow-up outcomes. In addition, we make an attempt to estimate the time investment and subsequent costs approximation of these procedures.

## 2 Methods

### 2.1 Patients and data selection

All six consecutive patients were included after informed consent. Demographics, procedure related and follow up data were obtained from the patient file. Five patients received a 3D-printed custom-made titanium anterior strut prosthesis, and one patient received a customized iliac allograft strut, which was personalized using 3D-printed milling guides. Four had a severe dystrophic spine due to NF type 1, one patient had Gorham's vanishing bone disease characterized by the destruction and absorption of in this case vertebral bodies (Nikolaou et al., 2014). The last pediatric patient had severe thoracic kyphoscoliosis of unknown cause, with previously failed growth guidance and posterior spinal fusion of the apical region, he received the allograft. An overview of patient characteristics is shown in Table 1. In patients where the posterior instrumentation had failed, the posterior instrumentation was first revised with strategical pedicle screw positioning so the pedicle screws would not interfere with the intended screw trajectories of the custom-made anterior prosthesis.

**Table 1:** Patient characteristics.

Patient	Age at surgery	Posterior instrumentation	Gender	Underlying disease	Spinal segments	Type of implant	Follow-up
1	16	Yes	M	NF1	C6 – Th11	Titanium	8 years
2	69	Yes	F	Vanishing bone	C5 – Th2	Titanium	7y 6m
3	20	Yes	M	NF1	Th2 – Th8	Titanium	4 years
4	15	Yes	F	NF1	Th8 – L2	Titanium	1y 6m
5	62	Yes	M	NF1	Th7 – L3	Titanium	1 year
6	6	Yes	M	Unknown	Th2 – Th10	Allograft	6 months

### 2.2 Anatomic data acquisition and clinical considerations

**Fig. 1:**
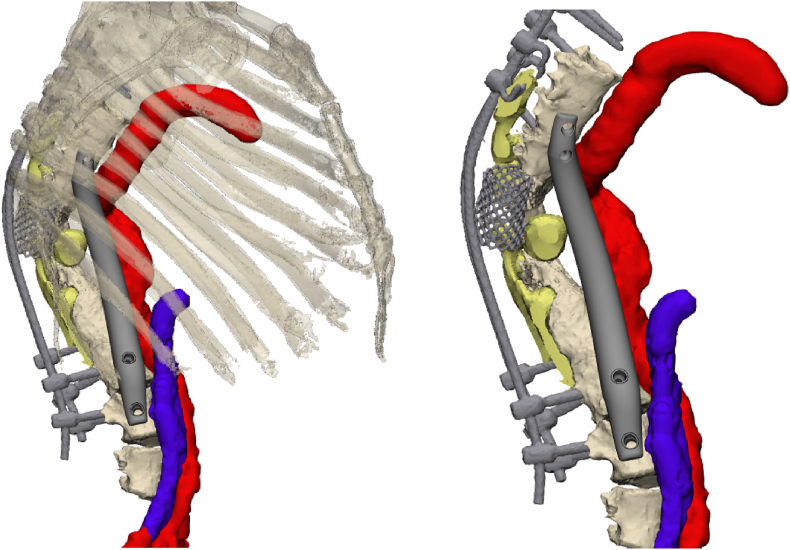
3D reconstruction of fused CT and MRI scans. Reconstructed from CT data are the anterior spine (Th6 – L4), the posterior instrumentation, and the mesh cage. Note that posterior elements and bone remnants surrounding the mesh cage were not segmented as these were very faint due to the dystrophic nature of the disease in combination with scattering on the CT. Reconstructed from MRI data are the dural ectasias, aorta and vena cava. The anterior prosthesis circumvents these critical structures.

Regular CT scans with a slice thickness of 1 mm and a metal artefact reduction protocol were acquired. The DICOM data was segmented using Mimics (version 26.0, Materialise, Leuven, Belgium) to create a virtual anatomical 3D model of the spine, ribs, posterior instrumentation and chest wall. If there were concerns about soft tissues surrounding the custom-made prosthesis, such as the aorta or vena cava, an additional CT angiography or MRI scan was obtained. These scans were fused with the initial CT scan in Mimics to segment the soft tissues within the same coordinate system (Fig. 1). The spine model was 3D printed in polyamide 12 (PA12) for ease of planning, multidisciplinary consultation and reference during surgery. Based on biomechanical and anatomical considerations, the appropriate vertebrae well above and below the affected segment were selected to anchor the buttresses at each end of the prosthesis. The buttress is designed in such a way that it supports the spine in the biomechanical axis, fits in the intervertebral space with limited endplate resection and integrates with the receiving bone though a trabecular surface.

### 2.3 Design and production of the titanium prosthesis

**Fig. 2:**
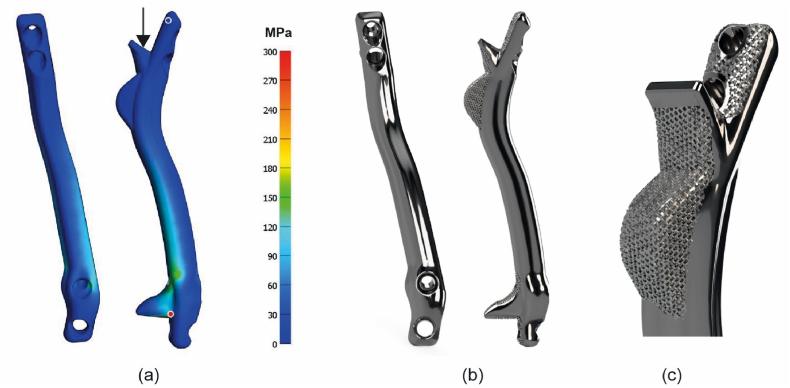
(a) Finite element analysis of the anterior spinal strut prosthesis, showing the distribution of von Mises stress in the design. The highest and lowest stress locations are indicated by the red and blue dot respectively. (b) Renders of the final custom-made anterior strut prosthesis. (c) Close up of the porous trabecular surface for osseointegration.

The design and manufacturing process has been described in more detail in prior research (Willemsen et al., 2019, 2022). We typically designed one standard and one 4 mm oversized prosthesis to allow for tight fit if necessary. The prosthesis consists of a bridging strut between two buttresses. Design considerations regarding the strut prostheses included amongst others locations and size of dural ectasias, location of the vena cava and bronchi and locations of pedicle screws in situ. The buttresses contain a 1 mm thick, 70 % porous titanium, trabecular-like surface layer for osseointegration with the receiving vertebral bone (Fig. 2b and c). One or two fixation screws are preplanned through each buttress which can be positioned with the help of sterilized, click-on drill guides, 3D printed in PA12. Considering the anatomical constrains, the bridging part between the buttresses has a cross-sectional area that is appropriate for weight bearing capacity required at the level of surgery. For instance, a cross-sectional area of 1 cm^2^, which is roughly the area equivalent of four 5.5 mm diameter rods has an approximated strength equivalent of eight 5.5 mm rods which should be sufficient for even the lower vertebrae levels. Moreover, a finite element analysis with a uniaxial load of 1000 N was performed for each implant to locate stress risers and adjust accordingly, see Fig. 2a. A minimal safety factor of 3, regarding the titanium alloy (Ti6Al4V) yield strength (800–1000 MPa) was used. As part of the recommended dossier building, a prospective risk analysis was performed by a multidisciplinary team. Potential risks were identified and mitigated where possible. The implants were manufactured externally in Ti6Al4V grade 23 using direct metal printing. Post processing steps included hot isostatic pressing, polishing and intermediate cleaning. Final (manual) cleaning, disinfection and autoclave sterilization of the implants was performed in-house in a certified facility.

### 2.4 Design and customization of the anterior allograft

**Fig. 3:**
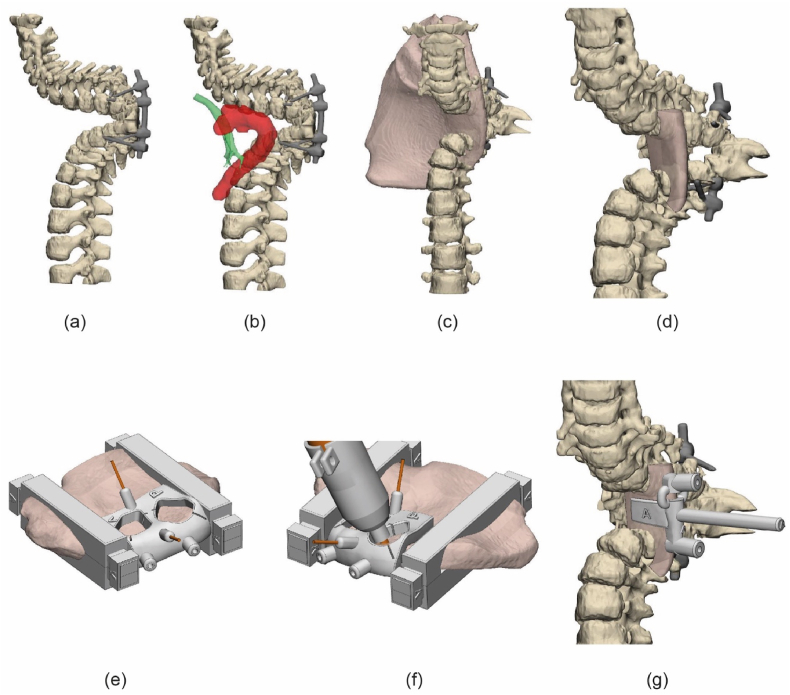
Planning and shaping of the allograft. (a) Segmented spine showing a 160° kyphosis. (b) The relative position of the trachea and aorta to the spine, reconstructed from CTA data. (c) The position of the iliac bone. (d) Final shape of the graft. (e) The allograft was stabilized by 3D-printed clamps and secured with K-wires and 4 mm holes were pre-drilled for later fixation. (f) Milling following the guide with the 3D printed adaptor. (g) The implant was positioned by using a custom-made clamping drill guide.

Due to the severe kyphoscoliosis (160°), the anterior allograft had to directly support the vertebral bodies and not the endplate. To take advantage of the biomechanical superiority of a tricortical graft, an iliac wing was used. Fig. 3 illustrates the methodology to shape the allograft. A 3D model of the iliac bone was created using a CT scan with bone thresholding in Mimics (Fig. 3a and b). The section of the allograft was determined to best fit between the receiving vertebrae with a minimal thickness of 5 mm and optimal use of the cortical surfaces (Fig. 3c and d). This structure was milled intraoperatively with a 6.0 mm carbide drill bit (DePuy Synthes, Raynham, MA, United States) by using a custom-made stabilizer clamp and milling guide that could follow the shape of the guide without touching it due to the 3D-printed adaptor, all designed using 3-matic (version 18.0, Materialise, Leuven, Belgium), Fig. 3e and f. A custom insertion clamp, which also served as a drilling guide for the screws (Fig. 3g), was also designed. All parts were 3D printed out of PA12 using selective laser sintering (SLM) and sterilized in-house.

### 2.5 Cost estimation of the implant

The time investment to build a case dossier in accordance with an appropriate quality management system (QMS), the hours spend on image processing, design, and consultation time with the surgeon were estimated retrospectively. Average production costs of the implants and allografts costs were retrieved from the manufacturer and bone bank quotations.

### 2.6 Surgical procedure

We used separate approaches for implants extending over two compartments e.g. thoracic and lumbar area. For thoracic procedures, one approach with partial rib resection was used. After level verification, the docking sides for the prosthesis were prepared and tested with PA12 dummies. A tunnel along the spine, through the diaphragm or thoracic apex, was created bluntly. Next, a 3D-printed PA12 trial prosthesis was used to confirm perfect fitting and determine the optimal implant size. The titanium prosthesis was inserted and temporarily fixated with K wires, using the 3D printed guides and 3D fluoroscopy was performed to confirm adequate positioning. The screw holes were drilled though the guides. A chest tube was applied for 1–2 days, and mobilization was without restrictions. Surgical duration, intraoperative blood loss and complications were extracted from the medical records.

## 3 Results

### 3.1 Titanium strut prostheses

**Fig. A1:**
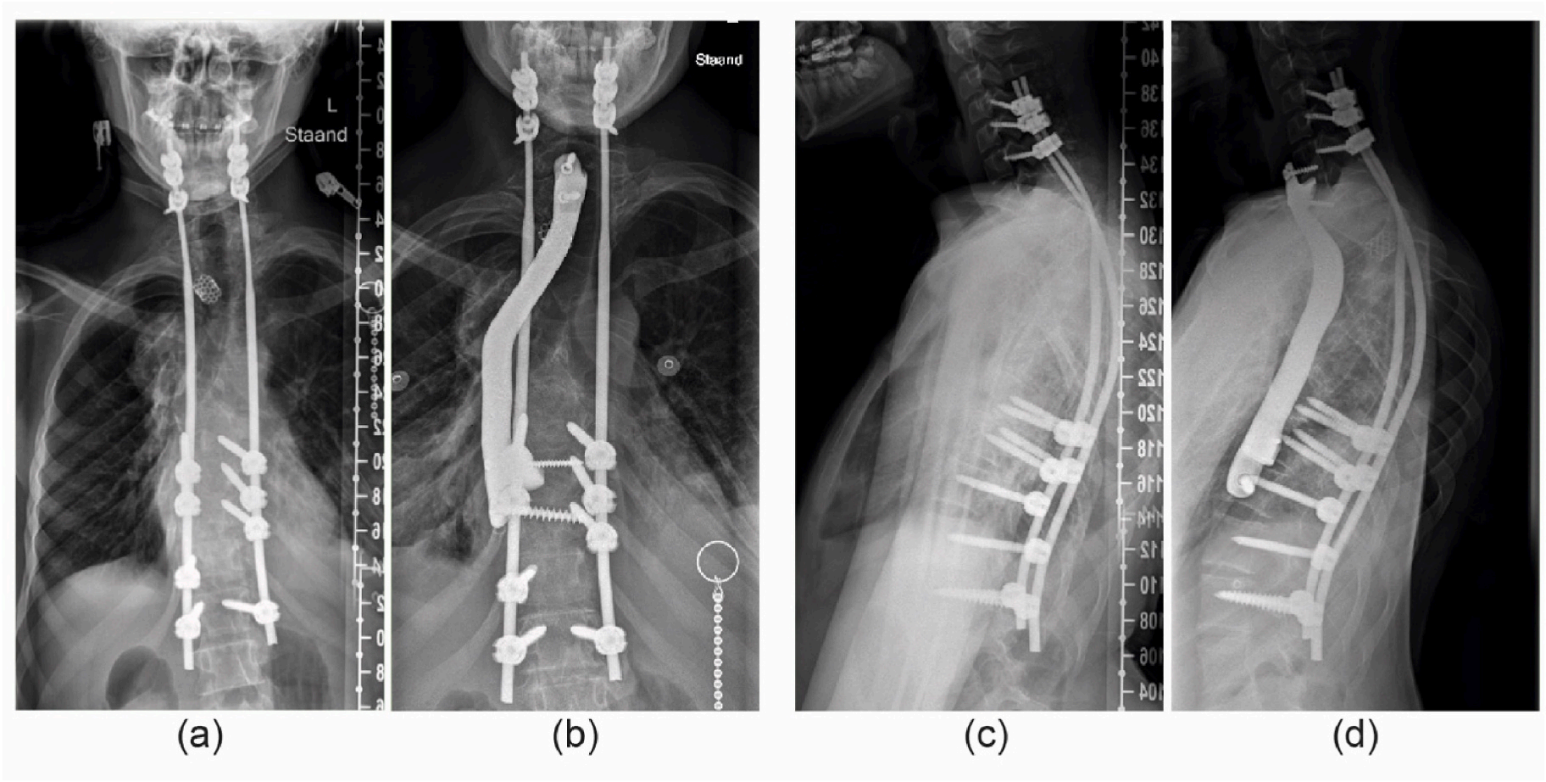
Patient 1; (a) Preoperative AP radiograph showing the situation after halo traction and posterior stabilization. (b) showing the anterior titanium strut prosthesis (C6-Th11). (c and d) Preoperative and postoperative lateral radiographs.

Case 1 a 16-year-old boy, with severe dystrophic kyphoscoliosis due to NF1 and was treated previously with posterior instrumentation. He presented with proximal junctional failure and neurological symptoms. With Halo traction neurological function returned, and he was first treated with posterior instrumentation. Because anterior support was lacking, a cervicothoracic orthosis was applied until he received the C6-Th11 anterior custom prosthesis after 6 months (Appendix Figure A1). During follow-up, reoperation was required to repair a fatigue failure of one of the posterior rods after 10 months. A postoperative CT revealed that, despite the prosthesis's minimal profile (10 mm), the right main bronchus was partially compressed, which may have contributed to his pulmonary function decline.

**Fig. A2:**
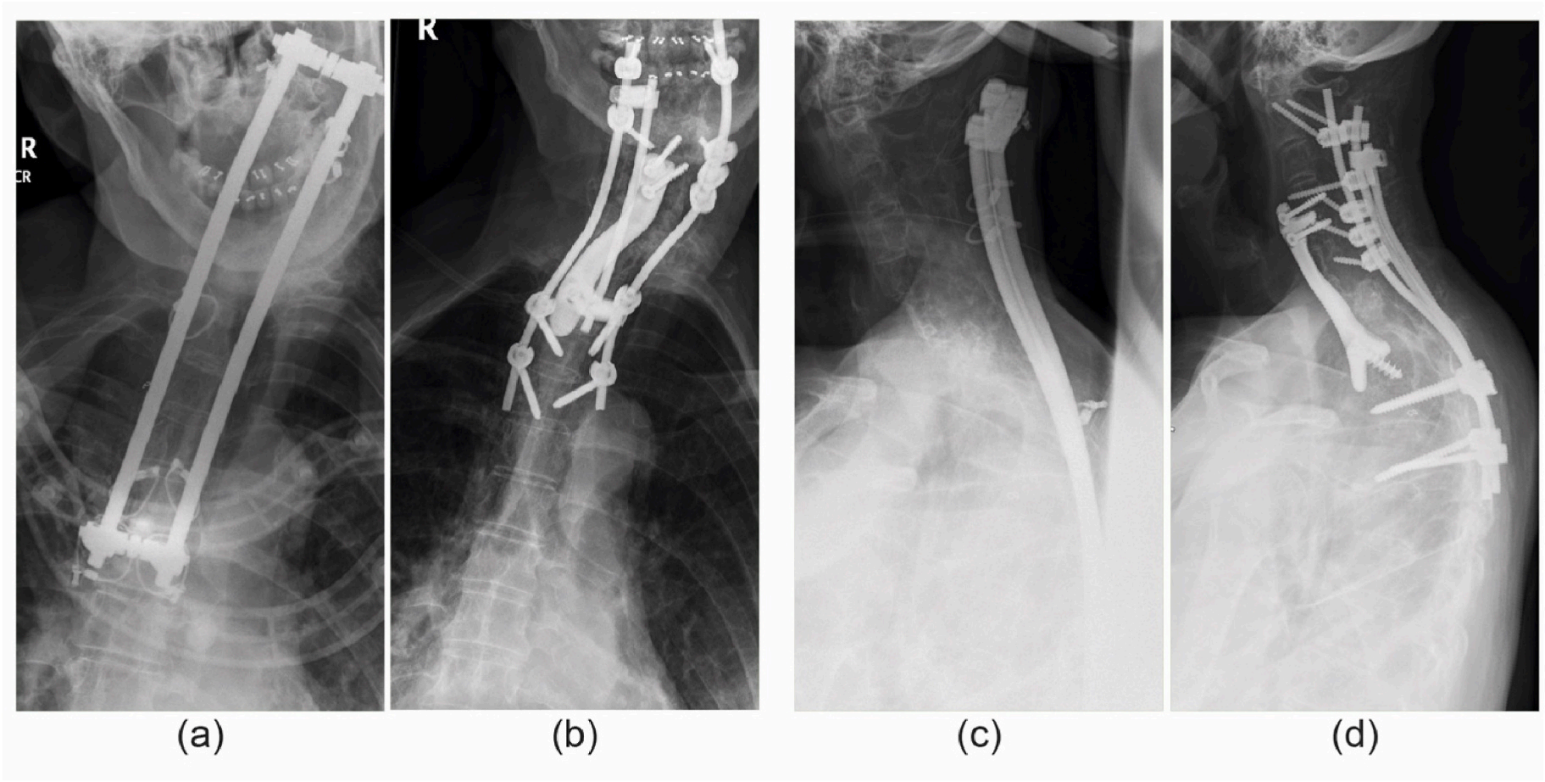
patient 2; (a) Preoperative AP radiograph showing the failed posterior fixation. (b) Postoperative AP radiograph showing the anterior titanium strut prosthesis (C5-Th2). (c and d) Preoperative and postoperative lateral radiographs.

Case 2 a 69-year-old female with a long history of vanishing bone disease of the cervical spine. She presented with an almost complete C7-Th1 dislocation with severe angulation and C7 resting on the first rib. Halo traction and posterior fixation was used as a temporary stabilization, which was successful to resolve the neurological deficit. She received a C5-Th2 prosthesis via one incision (Appendix Figure A2). No complications were recorded during 5 years of follow-up.

**Fig. A3:**
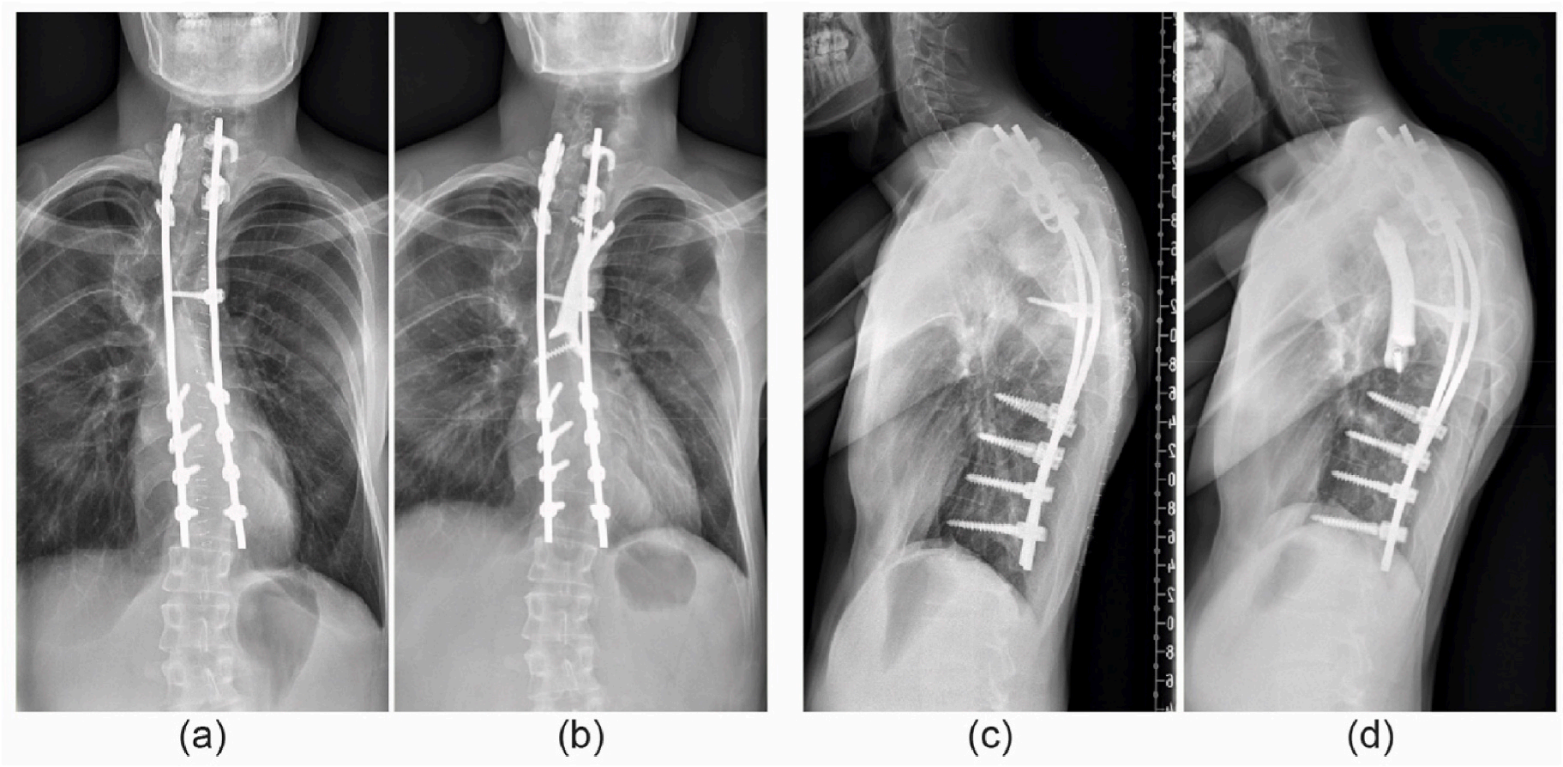
Patient 3; (a) Preoperative AP radiograph showing the revised posterior instrumentation in situ. (b) Postoperative AP radiograph showing the anterior titanium strut prosthesis (Th2-Th11). (c and d) Preoperative lateral radiograph.

Case 3 a 20-year-old male with dystrophic kyphoscoliosis due to NF1treated with posterior spinal fusion five years earlier. During follow-up the rods fractured, and the local thoracic kyphosis increased. To restore anterior support a Th2-Th8 prosthesis was inserted via a left thoracotomy with an uncomplicated follow-up (Appendix Figure A3). He presented 3.5 years later with neck pain that was possibly related to a neurofibroma. To exclude involvement of the prosthesis a CT scan was made which showed no signs of failure.

**Fig. 4:**
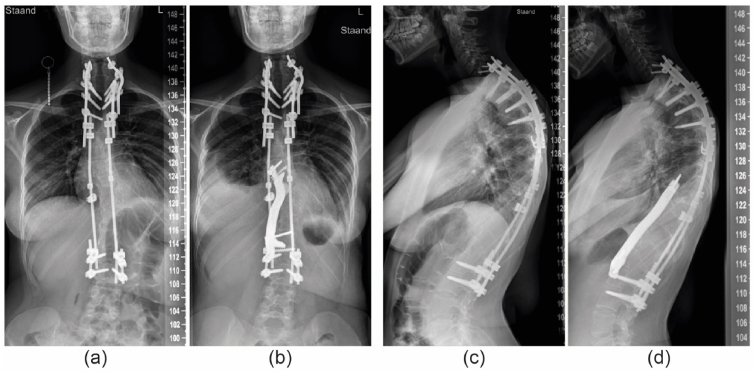
Patient 4; (a) Preoperative radiograph showing the posterior instrumentation and dystrophic thoracolumbar area. (b) after insertion of the anterior titanium strut prosthesis. (c) and. (d) lateral radiographs at the same timepoints.

Case 4 a 15-year-old female NF1 patient, treated since she was seven years old with posterior growth guidance for a syndromic, dystrophic scoliosis. Routine follow up revealed progressive disappearance and collapse of the anterior thoracolumbar spine. A Th8-L2 titanium prosthesis was placed to prevent complete spinal collapse (Fig. 2, Fig. 4). During 1.5 year of follow-up no complications were recorded.

**Fig. A4:**
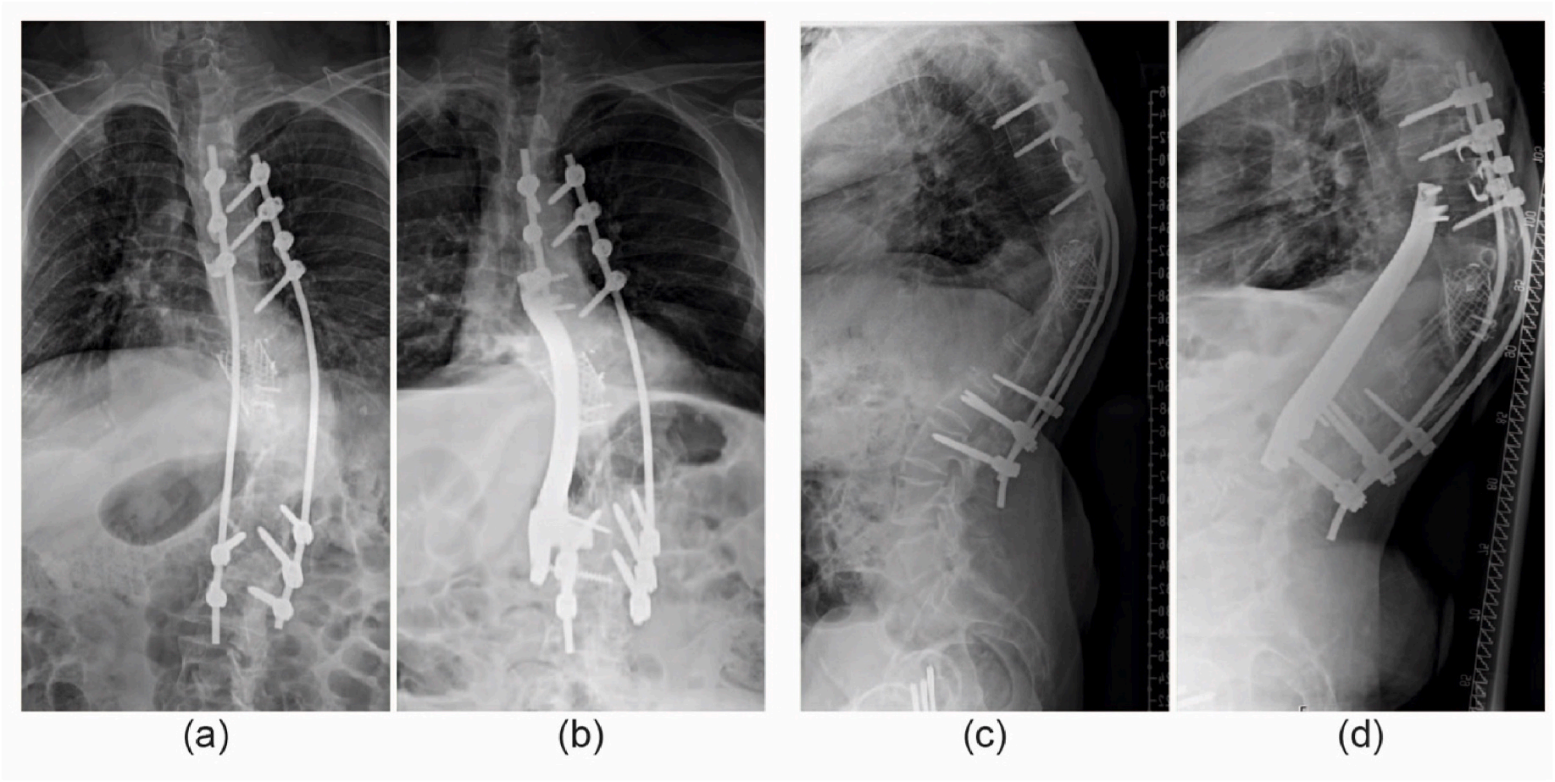
Patient 4; (a) Preoperative AP radiograph showing the posterior instrumentation and cage in situ. (b) Postoperative AP radiograph showing the anterior titanium strut prosthesis (Th7-L3). (c and d) Preoperative and postoperative lateral radiograph.

Case 5 a 62-year-old male NF1 patient, with previous posterior and anterior spinal fusion attempts, which could not prevent development of partial paraplegia as the anterior cage failed, and the deformity deteriorated. He received an anterior titanium strut prosthesis Th7-L3 (Appendix Figure A4). The procedure was complicated by a deep wound infection (Staphylococcus aureus) which could be treated with debridement and antibiotics. No signs of failure were observed during one year of follow-up.

For all cases, exposure and dummy fitting went smoothly as well as final insertion and fixation of the titanium prosthesis. In two cases the oversized version of the prosthesis was used to gain optimal internal stability. Postoperative radiographs confirmed the adequate position in all patients and in case 1 and 3 bone integration into the trabecular surface of the prosthesis was observed in CTs obtained for other reasons.

### 3.2 Structural allograft

**Fig. 5:**
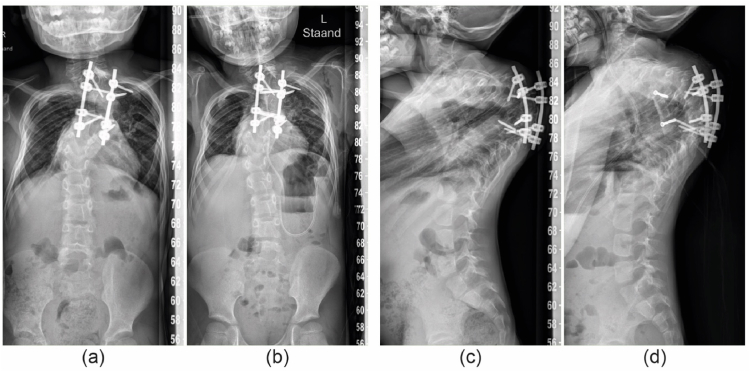
Patient 6; (a and b) Pre and post operative AP radiographs, (c and d) Pre and post operative lateral radiographs showing the 3D shaped allograft fixated with screws.

Case 6 a 6-year-old boy, with a severely progressive thoracic kyphoscoliosis. Despite extensive genetic screening and radiological workup, no cause of the spinal deformation was found. At the age of four, in situ short segment posterior fixation was performed to halt progression, which was complicated by temporary neuromonitor signal loss. During follow-up the kyphosis progressed up to 160°. An allograft strut was preferred over a titanium prosthesis to allow future remodeling and growth. A tricortical iliac allograft was chosen to support of the spine between Th2 and Th10 (Fig. 5). Especially myelum perfusion was a concern given the previous neuromonitoring signal loss and the need for ligation of some segmental vessels to mobilize the aorta. Exposure and insertion went uneventful without any neuromonitoring alerts. He was discharged after 6 days without restrictions and had no complications during the 6 months follow-up.

### 3.3 Surgical procedure

The mean OR time across all 6 patients was 219 min (range 116–287), the average blood loss was 850 mL (range 100–2500). The outcomes per patient are shown in Table 2.

**Table 2:** OR outcomes and complications.

Patient	OR time (minutes)	Blood loss (ml)	Complications
1	242	2500	Partial bronchial compression, posterior rod fracture
2	116	200	–
3	278	600	–
4	287	1200	–
5	167	500	Deep wound infection
6	226	100	–

### 3.4 Costs estimation

An estimation of the hours and costs of in-house development of the anterior spinal implants, including time spent on image processing, design, quality management dossier building and production is shown in Table 3. This overview indicates that most of the costs are initially on the design phase which declined with each case. External production costs of all titanium prosthesis were on average €1750, the costs of a hemipelvis allograft from a commercially available bone-bank were €2800.

**Table 3:** Time spent on different parts of the design process. Image processing involves DICOM export, segmentation exporting the STL files. Consultation hours is the time spent with the surgeon and multidisciplinary meetings.

Patient	Imaging modality	Image processing (hours)	Design (hours)	Consultation (hours)	QMS dossier building (hours)	Type of implant
1	CT	10	160	20	150	Ti6Al4V grade 23
2	CT	10	90	16	40	Ti6Al4V grade 23
3	CT	8	80	9	10	Ti6Al4V grade 23
4	CT	6	60	7	5	Ti6Al4V grade 23
5	CT + MRI	10	60	5	5	Ti6Al4V grade 23
6	CT + CTA	8	50	6	5	Allograft

#### 3.4.1 Dossier development and quality management system implementation

At the time of the first case described in this article (2019), formal dossier building was not required under the Medical Device Directive (MDD). However, with the impending implementation of the Medical Device Regulation (MDR), dossier development was initiated (see Table 3). Following this first case, a dossier format was established. For the second case, this patient-specific dossier was restructured into a more generalized ‘product dossier’, consolidating all types of anterior spinal constructs. This approach allowed for the maintenance of that core ‘product dossier’, with the addition of a brief patient-specific annex detailing case-specific considerations and modifications.

With the MDR coming into effect during the treatment of the third patient, time was invested in developing a comprehensive quality management system (QMS) based on ISO 13485 standards. This system enabled the integration of various implants, surgical guides, and procedures into distinct ‘product dossiers’, ensuring regulatory compliance while streamlining the design and manufacturing processes. This framework also facilitated the development of custom surgical guides for allograft fitting, as demonstrated in patient 6, which was made possible through an additional ‘product dossier’.

After the third case, dossier-building efficiency improved, leading to a progressive reduction in the time required for documentation and implant design (Table 3), an increase in implant quality, and a decrease in overall costs (design + production). Consequently, the in-house manufacturing pathway became increasingly beneficial, offering a cost-effective and efficient approach to producing high quality patient-specific spinal implants for complex patients.

## 4 Discussion

In this article we describe a customized treatment for anterior column failure in patients with very complex spines. Few other studies report on similar custom-made anterior spine prostheses; however, these are artificial vertebral bodies applied after a corpectomy and extending no more than 3 segments (Hu et al., 2022; Cao et al., 2023). In the current case series five patients received a titanium anterior strut prosthesis and one received a customized allograft, all extending multiple segments and often multiple compartments. In these instances, the prostheses only served as a strut, preventing collapse of the spinal column. Although the surgical procedures went smooth, three complications were recorded: fatigue fracture of the posterior instrumentation, impingement of a bronchus and deep wound infection. These complications underline the need for a strong implant that is customized to the patient anatomy and can be inserted fast with minimal exposure. During the follow-up up to 8 years, there were no signs of failure of the 3D-printed prosthesis itself. The follow-up period of the allograft was only 6 months, which does not allow conclusions on long term functionality although we do expect excellent replacement by remodeling (Saraph et al., 2005; Vaccaro and Cirello, 2002; Canalis, 1985). An obvious limitation in evaluating the efficacy of the anterior prosthesis is the difficulty to assess bony ingrowth in the porous titanium with the standard radiological follow up and even with CT scans (Duits et al., 2024). Metal artefacts such as beam hardening due to posterior instrumentation and the prosthesis itself make it nearly impossible to assess the bone-implant interface (White and Buckwalter, 2002). Palmquist et al. showed that even micro-CT does not suffice for this purpose if not calibrated with corresponding histology (Palmquist et al., 2017).

The costs of patient-specific approach as described in this case series are generally expected to be higher than off the shelf devices, especially for the first cases, due to the time invested in image processing, dossier building and one-off production costs. However, in challenging cases as described, the benefits of the customized approach seem to outweigh the costs, especially because off-the-shelf medical devices or conventional grafting procedures are unavailable or insufficient. Saraph et al. described the use of autologous anterior strut grafts using mostly iliac and fibula grafts. In their series however, they only included one patient with neurofibromatosis (Saraph et al., 2005). To be less dependent on a viable bone bed for engrafting, a vascularized rib or fibula graft, possibly supported by a femoral shaft allograft, is an option (Deen et al., 1999; Tobert et al., 2022). However, adjusting the shape of these allografts while remaining strong is difficult and studies describing this strategy concerned mostly chondrosarcoma resections in the thoracic spine where bone engraftment was not hampered by dural ectasias.

The most predominant costs of the custom-made implants were indeed the hours spent in dossier building and design. As expected, these costs lowered considerably (from 150 to 5 h per case for dossier building) due to the establishment of a standardized product dossier. Also design time was reduced a factor 3 due to optimizing the workflow. Time for Image processing however remained substantial, this is because the presence of posterior instrumentation results in metal artefacts that often require manual corrections (White and Buckwalter, 2002). The availability of high energy reconstructions of dual energy CT (DECT) scanners will reduce these costs by lowering metal artefacts and improving image quality (Bamberg et al., 2011). Also, deep learning algorithms have great potential to further reduce metal artefacts from DICOM images (Selles et al., 2024). Interestingly, the (material and production) costs of the titanium prosthesis itself are relatively low and considerably less than for example structural allografts.

Under the current MDR, hospitals in Europe are allowed to develop and apply custom devices if they comply with article 5.5 and Annex 1. An important side note to in-house development is Article 5c, which states that in-house development is only allowed if the target patient specific needs cannot be met at the appropriate level of performance by an equivalent device available on the market. In our opinion there are no medical devices available for reconstruction of the anterior spinal column which can be shaped and are strong enough to bridge multiple spinal segments. Most close come the expandable cage, however their use is typically limited to 3 vertebral levels (Cappelletto et al., 2020; Thongtrangan et al., 2003). Most important; these cages are relatively weak and cannot be shaped according to the patient specific anatomy which is especially needed to avoid impingement of vital structures in severe kyphotic thoracic spines.

## 5 Conclusion

In conclusion, custom-made prostheses appear to be a viable treatment option for multilevel anterior spinal support in severe kyphotic deformities of dystrophic spines. No implant failure has been observed up to 5-year follow-up. We demonstrate the legal possibility and (cost) efficacy of using 3D-customized anterior spinal. A prerequisite for this approach is the availability of an in-house, dedicated 3D lab that has the appropriate technical knowledge, can invest in building a standardized workflow and dossier format and has the manpower for regular consultation and multidisciplinary panel discussions.

## Author contributions

JM, MR, KW, HW, TS, MK: Conceptualized the study; JM, MR, KW, MK: Co-designed the custom-made prosthesis and allografts; TS, MK: Clinical application; JM, MR, TS, MK: data collection; JM, MR, KW, HW, TS, MK: Wrote, edited and reviewed the manuscript. All authors have read and approved the final submitted manuscript.

## Funding

The research for this paper was partially financially supported by the PRosPERoS-II project, funded by the 10.13039/100013276Interreg VA 10.13039/501100011878Flanders – the Netherlands program, CCI grant no. 2021TC16RFCB041.

## Declaration of competing interest

The authors declare the following financial interests/personal relationships which may be considered as potential competing interests: Outside this work: Speaker's fee: MBA Healthcare, J&J; Board of directors: EUROSPINE: TS If there are other authors, they declare that they have no known competing financial interests or personal relationships that could have appeared to influence the work reported in this paper.
